# miR-7977 inhibits the Hippo-YAP signaling pathway in bone marrow mesenchymal stromal cells

**DOI:** 10.1371/journal.pone.0213220

**Published:** 2019-03-05

**Authors:** Masahiro Yoshida, Hiroto Horiguchi, Shohei Kikuchi, Satoshi Iyama, Hiroshi Ikeda, Akari Goto, Yutaka Kawano, Kazuyuki Murase, Kohichi Takada, Koji Miyanishi, Junji Kato, Masayoshi Kobune

**Affiliations:** 1 Department of Hematology, Sapporo Medical University School of Medicine, Sapporo, Hokkaido, Japan; 2 Department of Medical Oncology, Sapporo Medical University School of Medicine, Sapporo, Hokkaido, Japan; Mayo Clinic Minnesota, UNITED STATES

## Abstract

We and others have demonstrated that various abnormalities of the bone marrow (BM) mesenchymal stromal cells (MSCs) such as aberrant cytokine expression, abnormal hedgehog signaling, and impaired miRNA biogenesis are observed in patients with acute myeloid leukemia (AML). However, underlying mechanisms to induce the dysfunction of BM MSCs have not yet been clarified. We previously showed that AML cells release abundant exosomal miR-7977, which, in turn, enters BM mesenchymal stromal cells (MSCs). However, the precise function of miR-7977 is not known. In this study, we performed transduction of a miR-7977 mimic into MSCs, compared transcriptomes between control-transduced (n = 3) and miR-7977-transduced MSCs (n = 3), and conducted pathway analysis. The array data revealed that the expression of 0.05% of genes was reduced 2-fold and the expression of 0.01% of genes was increased 2-fold. Interestingly, approximately half of these genes possessed a miR-7977 target site, while the other genes did not, suggesting that miR-7977 regulates the gene expression level directly and indirectly. Gene set enrichment analysis showed that the gene sets of Yes-associated protein 1 (YAP1) _up were significantly enriched (p<0.001, q<0.25), suggesting that miR-7977 modulates the Hippo-YAP signaling pathway. Visualization of pathway and network showed that miR-7977 significantly reduced the expression of Hippo core kinase, STK4. miR-7977 inactivated the Hippo-YAP signaling pathway as proven by GFP-tagged YAP nuclear trans localization and TEAD reporter assay. The miR-7977-transduced MSC cell line, HTS-5, showed elevated saturation density and enhanced entry into the cell cycle. These results suggest that miR-7977 is a critical factor that regulates the Hippo-YAP signaling pathway in BM-MSCs and may be involved in the upregulation of leukemia-supporting stroma growth.

## Introduction

We and others have revealed that various abnormalities of the bone marrow (BM) environment such as stromal dysfunction [1], aberrant cytokine expression [2] and impaired microRNA (miRNA) biogenesis[3] are observed in patients with myelodysplastic syndrome (MDS) and acute myeloid leukemia (AML). Moreover, these abnormalities may be involved in the development of MDS/AML as shown by studies using mesenchymal progenitor-specific knockout mice that demonstrated impaired miRNA biogenesis in BM mesenchymal stromal cells (MSCs) and the development of MDS [4].

Recently, underlying mechanisms involved in BM stromal dysfunction, genomic methylation [5, 6] and immunological disturbance [7] of MSCs have been identified. More recently, we have focused on extracellular vesicles including exosomes, which are abundant in the BM microenvironment [8]. Further, we have shown that exosomal miR-7977 was highly released from AML cells and was taken up by BM MSCs. Poly(rC) binding protein 1 (PCBP1) was predicted to be a target gene of miR-7977 by an online database for miRNA target prediction using miRDB (http://mirdb.org/cgi-bin/search.cgi?searchType=miRNA&full=mirbase&searchBox), and reductions in PCBP1 mRNA and protein were confirmed in MSCs after miR-7977 transfer. PCBP1 is involved in splicing, and the expression levels of several genes including stem cell factor (SCF), angiopoietin-1 (ANGPT1) and Jagged-1 (JAG1) were also decreased [2]. Hence, the exosomal miR-7977 could play an important role in the disturbance of normal hematopoiesis in MDS/AML.

However, in general, a miRNA has multiple targets. In fact, miRDB predicted 633 targets of genes although the target rank and target score of PCBP1 were the highest. Moreover, the function of an exosomal miRNA could be different according to the type of cells. One possibility is that the efficiency of exosomal transfer could differ depending on surface antigens, ligands and receptors on exosomes and cells [9]. Another possibility is that certain unknown long noncoding RNAs such as HOTAIR or PCBP1-1:1 may sponge several miRNAs [10]. Therefore, the precise targets and function of miR-7977 in BM MSCs have not been clarified.

In the present study, a control vector or miR-7977mimic was transfected into BM MSCs. Subsequently, alteration of transcriptome was analyzed, and pathway analysis was conducted. We found that several components of the Hippo-YAP signaling pathway were significantly enriched. Hence, we focused on the Hippo-YAP signaling pathway and analyzed biological alterations of miR-7977-transferred MSCs.

## Materials and methods

### Reagents, human BM MSCs and HTS-5

Rabbit monoclonal anti-serine/threonine-protein kinase 4 (STK4) antibody (Ab) (ab51134), mouse monoclonal anti-STK4 Ab (ab57836), anti-YAP1 Ab (ab52771) anti-YAP1 Ab (phospho Ser127; ab76252) and anti-NF2/Merlin Ab (ab88957) were purchased from Abcam (Tokyo, Japan). Human BM CD34+ cells and three different lot numbers of MSCs from healthy volunteers (HV) were purchased from AllCells, LLC (Toronto, Canada) (HV-derived MSCs #1, #2, #3). The MSCs were cultured in MSCGM hMSC basal medium with the addition of supplements from the MSCGM hMSC SingleQuot Kit (Lonza Japan Ltd, Tokyo, Japan). The establishment of human hTERT-BM mesenchymal cell clone, HTS-5, was reported previously [11, 12]. HTS-5 cells were cultured in Dulbecco's Modified Eagle Medium (DMEM) containing 10% heat-inactivated fetal calf serum (FCS) (Gibco BRL), 2 mM/L L-glutamine, 0.1% penicillin (100 U/mL) and streptomycin (100 mg/mL) at 37°C under 5% CO_2_ in a humidified atmosphere [11–13].

### Analysis of mRNA and miRNA expression

For reverse transcription, total RNA was prepared from cells using the Trizol reagent according to the manufacturer’s instructions (Invitrogen, Waltham, MA). Total RNA (1 μg) was reverse transcribed with the SuperScript VILO cDNA Synthesis Kit (Invitrogen), RT^2^ First Strand Kit (QIAGEN, Hilden, Germany) and miScript ‖ RT Kit (QIAGEN) for Taqman PCR, SYBR Green PCR and miScript SYBR Green PCR (QIAGEN), respectively. Primer set IDs for real-time SYBR Green PCR and miScript Primer assay are indicated in S1 Table. Quantitative real-time PCR (qRT-PCR) was performed in triplicate using the ABI PRISM7300 Sequence Detection System (Applied Biosystems, Waltham, MA) in a 25 μL reaction volume.

### Transfection of siRNA or miRNA mimics into MSCs

miRNA mimics (QIAGEN) were utilized to confirm the target of each miRNA (S2 Table). Transfection of siRNA or miRNA mimic (5 nM for primary MSC and 20 nM for HTS-5) was conducted using Lipofectamine LTX Reagent or Lipofectamine RNAiMAX Transfection Reagent (Life Technologies) or HiPerFect Transfection Reagent (QIAGEN). The mRNA expression was analyzed by qRT-PCR 48–72 hrs after miRNA transfer.

### Reporter assay using luciferase and GFP-tagged YAP1

Reporter plasmids that expressed luciferase with 8 TEAD (TEA Domain Transcription Factor) binding sites (8xGTIIC-luciferase, Plasmid#34615) or YAP1 (GFP-tagged)-pCMV6-AC-GFP (RG225864) were purchased from Addgene (Cambridge, MA) or OriGene Inc. (Rockville, MD), respectively. Expression plasmid of human miR-7977 (MIR7977-MiRNA) and empty vector control (pCMV-MIR) were purchased from OriGene Inc. MSCs or HTS-5 cells were transfected with 2 μg of the 8xGTIIC-luciferase, YAP1 (GFP-tagged)-pCMV6-AC-GFP, control pMirTarget plasmid (pCMV-MIR) or pCMV-MIR-7977 using Lipofectamine LTX transfection reagent (Life Technologies). After transfection, the cells were incubated at 37°C under 5% CO_2_ for 48 hrs, and then luciferase activities were measured according to the manufacturer’s recommendation (Promega, Madison, WI) using a lumat LB 9507 luminometer (Berthold Technology, Bad Wildbad, Germany). Effects of miR-7977 are presented as the ratio of the normalized value to the light emission observed in the cells transduced with the control pCMV-MIR vector. Nuclear localization of GFP-tagged YAP1 was visualized by using a Biozero BZ-8000 laser scanning microscope (KEYENCE, Laboratories, Tokyo, Japan) as reported previously [14].

### Analysis of array data

The mRNA was analyzed by Human Gene 2.0ST Array and compared. A scattered plot demonstrated the total RNA level in control MSCs and miR-7977-transduced MSCs. CEL data were normalized by the rma method provided from Bioconductor (R commander 3.4.1). Dendrogram and heatmap were made by using the amap and gplots package. The genes shown were selected when the expression level significantly differed between the control and miR-7977-transduced MSCs. The gene expression level in the heatmap was expressed by intensity-based Z-score.

### Gene set enrichment analysis and network analysis

Gene set enrichment analysis (GSEA) was conducted using open source software GSEA 3.0 (http://software.broadinstitute.org). As gene sets database, h.all.v6.0.symbol.gmt [Hallmarks], c2.cp.biocarta.v6.0.symbols.gmt [Curated], c2.cp.kegg.v6.0.symbols.gmt, c2.cp.reactome.v6.0.symbols.gmt [Curated] and c6all.v6.0.symbols.gmt [Oncogenic signature] were used. The pathways showing NOM p-val (p-value) < 0.05 or false discovery rate (FDR) q-value < 0.25 were considered as significant. The significant pathway was rolled out as a network or a KEGG (Kyoto Encyclopedia of Genes and Genomes) pathway map using Cytoscape software version 3.6.1 (https://cytoscape.org/download.html). The fold change of gene expression [Log2(miR-7977 transduced MSCs/ Control transduced MSCs)] was superimposed on the molecular interaction network.

### Statistical analysis

Each data set was first evaluated for normality of distribution by the Komolgorov-Smirnov test to decide whether a non-parametric rank-based analysis or a parametric analysis should be used. The significance of differences between groups was assessed by ANOVA, followed by Dunnett’s multiple comparison tests. Results are expressed as the mean ± standard deviation (SD). The significance of differences was assessed by either the Student’s t-test or the Mann-Whitney U-test, and a p-value < 0.05 was considered as statistically significant.

## Results

### Alteration of transcriptome after miR-7977 transfer

**Fig 1:**
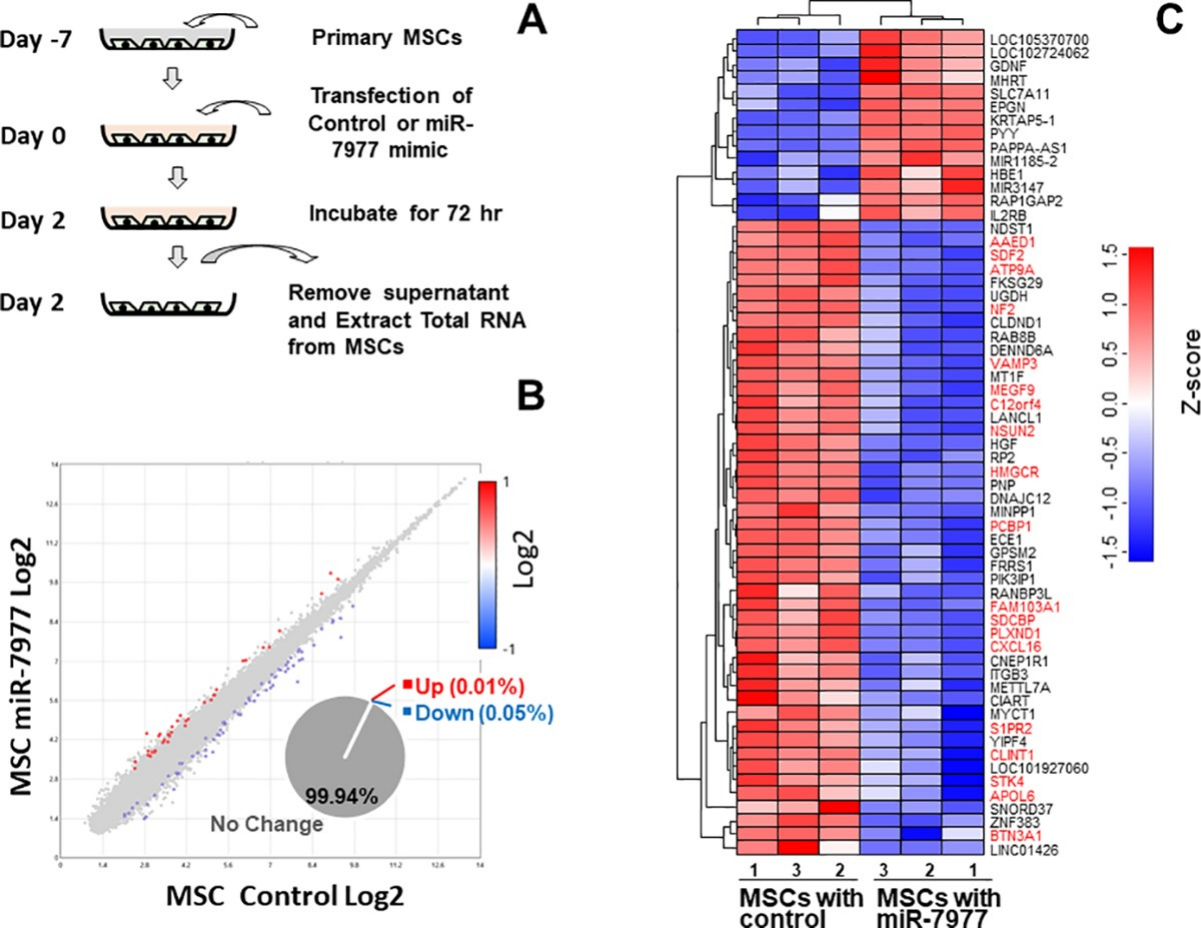
Comparative analysis of mRNA expression between control- and miR-7977-transduced MSCs. (A) Transfection of control or miR-7977 mimic into MSCs. 5 nM of miR-7977 mimic was used for transfection. After 72 hrs, total RNA was prepared for transcriptome analysis. (B) The mRNA was analyzed by Human Gene 2.0ST Array and compared. Scattered plot demonstrates the total RNA level in control MSCs and miR-7977-transduced MSCs. (C) Cluster analysis of mRNA expression in MSCs. The gene symbols that have the target sites predicted by miRDB are indicated by red ink. CEL data were normalized by the rma method provided by Bioconductor (R commander 3.4.1). Dendrogram and heatmap were made by using the amap and gplots package. The genes shown were selected when the expression level significantly differed between control and miR-7977-transduced MSCs [control (n = 3) vs. miR-7977 (n = 3), P = 0.023]. The gene expression level in the heatmap was expressed by intensity-based Z-score.

To gain insight into the precise function of miR-7977 in BM MSCs, control or miR-7977mimic was transferred into BM MSCs (Fig 1A). Subsequently, the transcriptome was analyzed by using Human Gene 2.0ST Array. Scattered plot demonstrated that the expression level of multiple genes showed a 2-fold change after miR-7977 transfer into BM MSCs. The array data revealed that 0.05% of genes showed a 2-fold decrease in expression and 0.01% of genes showed a 2-fold increase in expression (Fig 1B). To visualize the change in gene expression, cluster analysis was conducted. As shown in Fig 1C, the type of changes in gene expression was divided into two clusters. One cluster was downregulated, and the other cluster was upregulated. Detail information of downregulated genes were shown in S3 Table. The upregulated genes did not have any miRDB predicted target site of miR-7977 in their mRNA, suggesting that their expression levels were indirectly elevated by miR-7977. Importantly, in the downregulated cluster, some of the genes possessed the target sites of miR-7977 (Fig 1C), while other genes did not possess any target sites of miR-7977. These results indicated that the effect of miR-7977 appears to be complicated and the effect should be comprehensively analyzed.

### Pathway analysis

**Fig 2:**
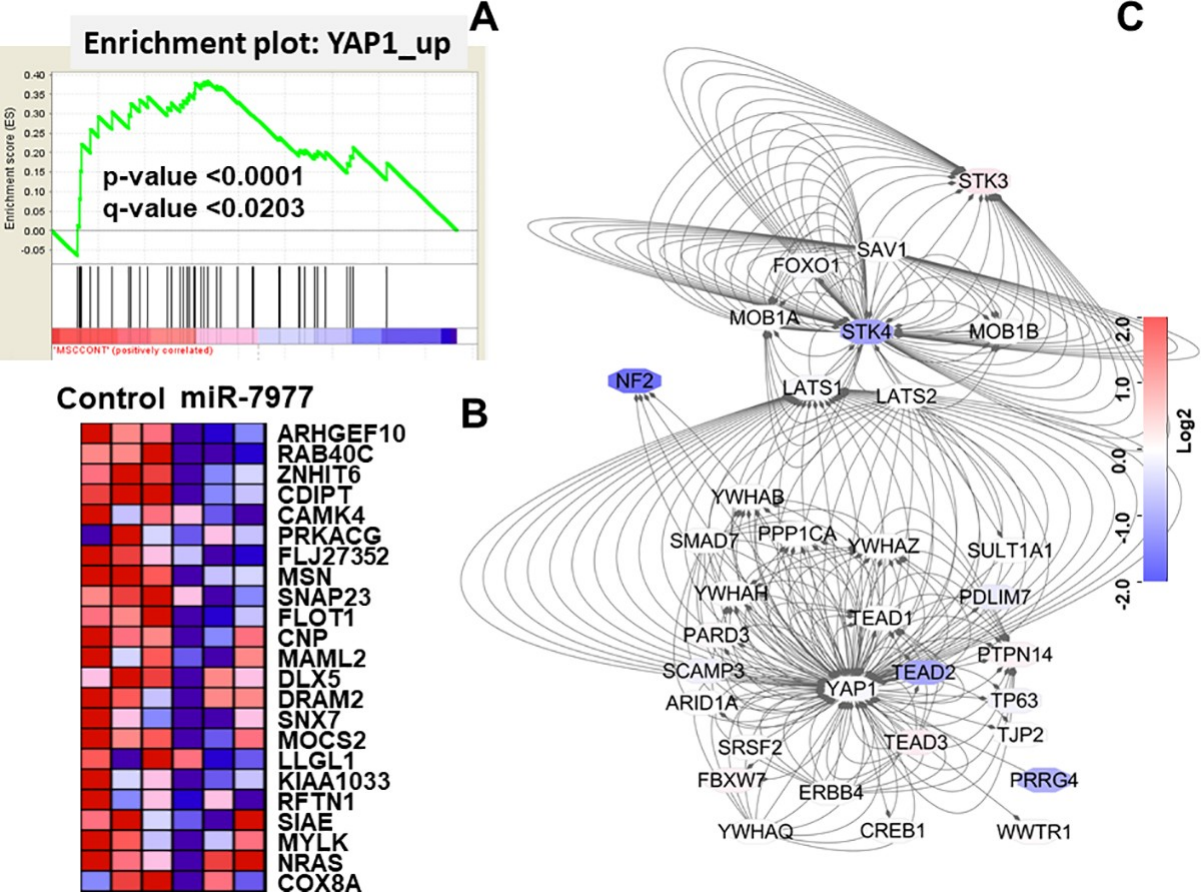
Array data were analyzed by GSEA and Cytoscape version 3.6.1. (A) Enrichment plots from GSEA are shown. The panel was obtained by GSEA using the gene set database, c6all.v6.0.symbols.gmt [Oncogenic signature]. (B) The core enrichment of genes was visualized as heatmap. Left side showed control MSCs and Right side showed miR-7977-transduced MSCs. (C) The expression of each gene associated with YAP1 and tight junction was visualized as a molecular interaction network made by Cytoscape software. Gene symbols are overlaid on each node and interactions between genes are shown as dotted lines with an arrow (edge). The fold change of gene expression [Log_2_(miR-7977 transduced MSCs/ Control transduced MSCs)] was superimposed on the molecular interaction network.

We subsequently conducted pathway analysis using GSEA 3.0. Many pathways were significant (NOM p-val<0.00000001, S4 Table). Among them, the FDR q-value of the YAP1_UP-related Hippo signaling pathway, KEGG_VASOPRESSIN_REGULATED_WATER_REABSORPTION and KEGG_SNARE_INTERACTIONS_IN_VESICULAR_TRANSPORT was less than 0.25, indicating that these pathways were strongly significant (Table 1). However, when the maps of KEGG pathway superimposed of fold change of genes was assessed, the alteration of expression of each component of genes was inconsequence. Some of genes were upregulated and others were downregulated (S1 Fig and S2 Fig). Conversely, when core enrichment genes of YAP1_UP were evaluated, the gene expressions of downstream of YAP1 was consistently decreased (Fig 2A and 2B). Because core enrichment genes of YAP1_UP consist almost entirely of downstream of Hippo signaling, the upstream of Hippo signaling was reconstituted and the molecular interaction network of Hippo signaling-associated genes was visualized by using Cytoscape software. The fold changes of gene expression (miR-7977-transduced/control-transduced MSCs) were superimposed as a heatmap on the molecular interaction network. The expression levels of several genes were significantly decreased such as neurofibromin 2 (NF2) and Hippo core kinase: serine/threonine kinase 4 (STK4). From this interaction network, STK4 but not STK3 could be mainly involved in Hippo signaling pathway via LATS (Large Tumor Suppressor Kinase) 1/2 in BM MSCs. Moreover, as Hippo signaling targets, YAP1 but not WWTR1 (TAZ: transcriptional co-activator with PDZ-binding motif) could mainly act in combination with TEAD families in BM MSCs. Inconsistent with previous report [15], NF2 appeared to be directly associated with YAP by LAT1/2-independent manner. These results suggested that miR-7977 inactivates Hippo signaling via reduction of upstream core kinase. However, we thought that more detail analyses regarding expression and association of SKT4 and NF2 in Hippo signaling should be required.

**Table 1:** Summary of results of GSEA showing p-value <0.00001 and q-value <0.25.

GENE SET NAME	NES	NOM p-value	FDR q-value
YAP1_UP	1.5068	0.0000	0.2027
KEGG_SNARE_INTERACTIONS_IN_VESICULAR_TRANSPORT	-1.6243	0.0000	0.2333
KEGG_VASOPRESSIN_REGULATED_WATER_REABSORPTION	-1.8425	0.0000	0.0520

Gene sets enriched in phenotype of control transduced MSCs as compared with miR-7977 transduced MSCs. YAP1_UP: Genes up-regulated in MCF10A (Breast cancer) cells over-expressing YAP1 gene. NES: normalized enrichment score.

### Quantitative analysis of STK4 and YAP1

**Fig 3:**
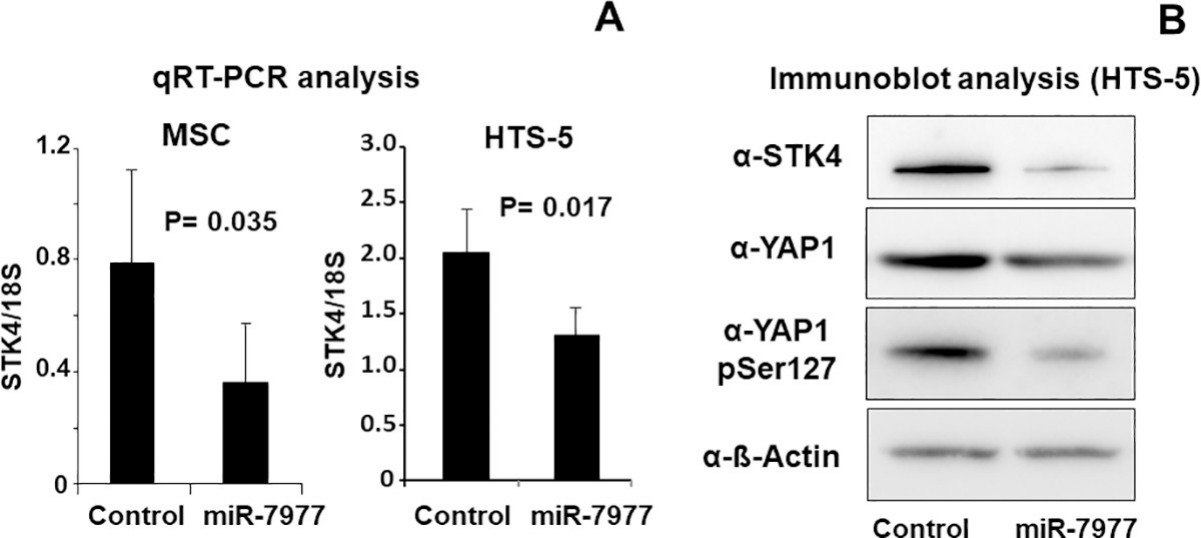
Transduction of miR-7977 reduced the level of STK4 and YAP1. (A) qRT-PCR for STK4 was conducted after transduction of control or miR-7977 for primary MSCs (5 nM) and HTS-5 (20 nM). The left panel shows the results in MSC (n = 5), and the right panel shows the results in the HTS-5 mesenchymal cell line (n = 3). (B) Immunoblot analysis of Hippo signaling pathway after transfer of control siRNA or miR-7977 into HTS-5. Anti-STK4 Ab, anti-YAP1 and anti- phospho-YAP1 Ser127 were used. As internal standard, anti-β-actin was used.

We next confirmed the expression levels of positive Hippo signaling regulators, STK4 and NF2. The expression levels of STK4 and NF2 were confirmed by real-time PCR. As shown in Fig 3A, miR-7977 transfer significantly reduced the level of STK4 mRNA in primary BM MSCs and in the HTS-5 mesenchymal cell line. Moreover, the expression of STK4 protein and phosho-YAP1 serine 127 (cytoplasmic form) was also reduced after miR-7977 transfer although whole level of YAP1 protein was not changed (Fig 3B and S3 Fig). The expression level of STK4 protein was remarkably reduced as compared with reduction of STK4 mRNA after miR-7977 transfer. In this regard, we analyzed the target sites of miR-7977 in STK4 mRNA by MR-microT software provided by DIANA tool (http://diana.imis.athena-innovation.gr/DianaTools/index.php?r=site/index). The eight target sites were detected and shown in Table 2. The seven target sites existed in 3’UTR (untranslated region) and one target site existed in cording sequence (CDS). It was revealed that miRNA target sites located in CDS effectively inhibit translation [16]. Hence, the expression level of STK4 protein may be considerably reduced. Regarding NF2, although the level of NF2 mRNA in primary BM MSCs was significantly reduced (S4 Fig), the reduction of NF2 protein was marginal (S4 Fig). These results indicated that miR-7977 could reduce the STK4 level and inactivate Hippo signaling.

**Table 2:** miR-7977 target prediction was conducted using MR-microT software provided by web site of DIANA tool.

	custom ENST0000037280647 (STK4)		Total score 0.997906
miR-7977 target region	Binding Type	Transcript Position	Score
UTR3	6mer	135–143	0.00304507
UTR3	8mer	280–288	0.081285224
CDS	7mer	625–633	0.01236068
UTR3	7mer	1612–1620	0.008625239
UTR3	7mer	2338–2346	0.006710955
UTR3	7mer	2474–2482	0.00290708
UTR3	7mer	3292–3300	0.003534501
UTR3	7mer	4096–4104	0.008759912

UTR3: Three prime untranslated region; CDS: cording sequence

### Evaluation of Hippo signaling by reporter assay

**Fig 4:**
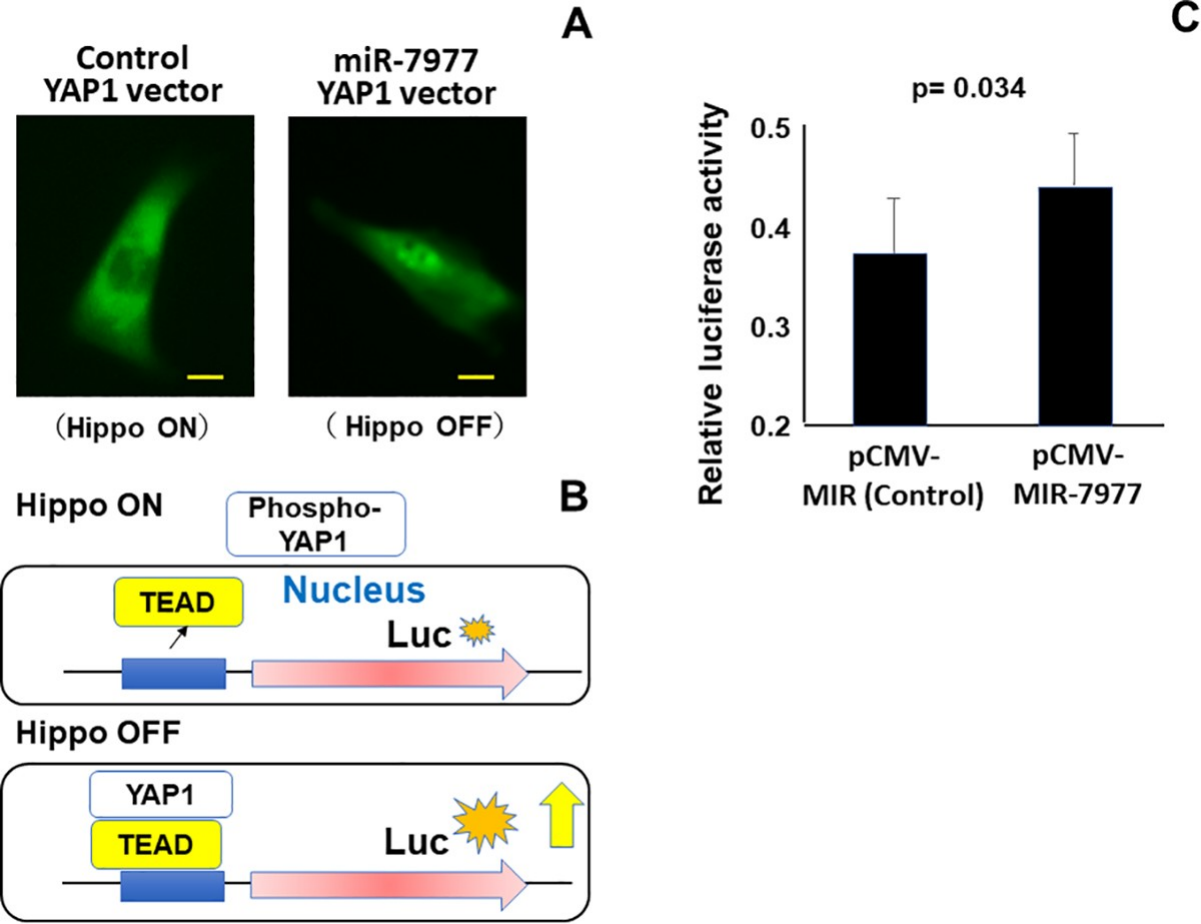
Transduction of miR-7977 induce nuclear localization of YAP1 and inactivated Hippo-YAP signaling via transcription factor TEAD. (A) Nuclear localization of GFP-tagged YAP1 after transduction of YAP1 (GFP-tagged)—pCMV6-AC-GFP into HTS-5. Subsequently, control or 20 nM miR-7977 mimic was transfected, and the cells were visualized. Left panel: control siRNA transfer. Right panel: miR-7977 transfer. Scale = 10 μm. (B) Schematic diagram of luciferase assay to evaluate the interaction of TEAD and YAP1. (C) Transduction of pCMV-MIR-7977 and 8xGTIIC-luciferase into the HTS-5 stromal cell line and analysis of luciferase activity 2 days after transduction. Luciferase activity was normalized to the activity of an internal control (renilla luciferase). *P < 0.05, pCMV-MIR (control, n = 8) vs. pCMV-MIR-7977 (n = 8) (Student’s *t*-test). Data represent three independent experiments, each done in octuplicate.

To confirm the inactivation of Hippo signaling, we conducted two reporter assays. First, GFP-tagged YAP1 was transduced into HTS-5 and subsequently, control or miR-7977 mimic was transduced into GFP-tagged YAP1-expressing HTS-5. As shown in Fig 4A, GFP-tagged YAP1 was present in the cytosol of control-transduced MSCs. GFP-tagged YAP1 was localized in the nucleus and cytosol of miR-7977-transduced MSCs, indicating that miR-7977 induced the Hippo OFF condition. As shown in Fig 2C, the transfer of miR-7977 slightly altered the expression level of transcription factor, TEAD2 and TEAD3. It is possible that the level of TEAD expression are associated with Hippo signaling pathway (Fig 4B). We employed 8xGTIIC-luciferase reporter plasmid which has eight binding sites of the TEAD. TEAD binds to nuclear localized YAP1 and transcribes several genes involved in entry into the cell cycle and apoptosis. As shown in Fig 4C and S5 Fig, luciferase activity was significantly increased when the miR-7977-expression vector was co-transfected into HTS-5. These results strongly suggest that miR-7977 inactivates Hippo-YAP1 signaling which is possibly associated with STK4 reduction.

### Analysis of the function of miR-7977-transduced mesenchymal cells

**Fig 5:**
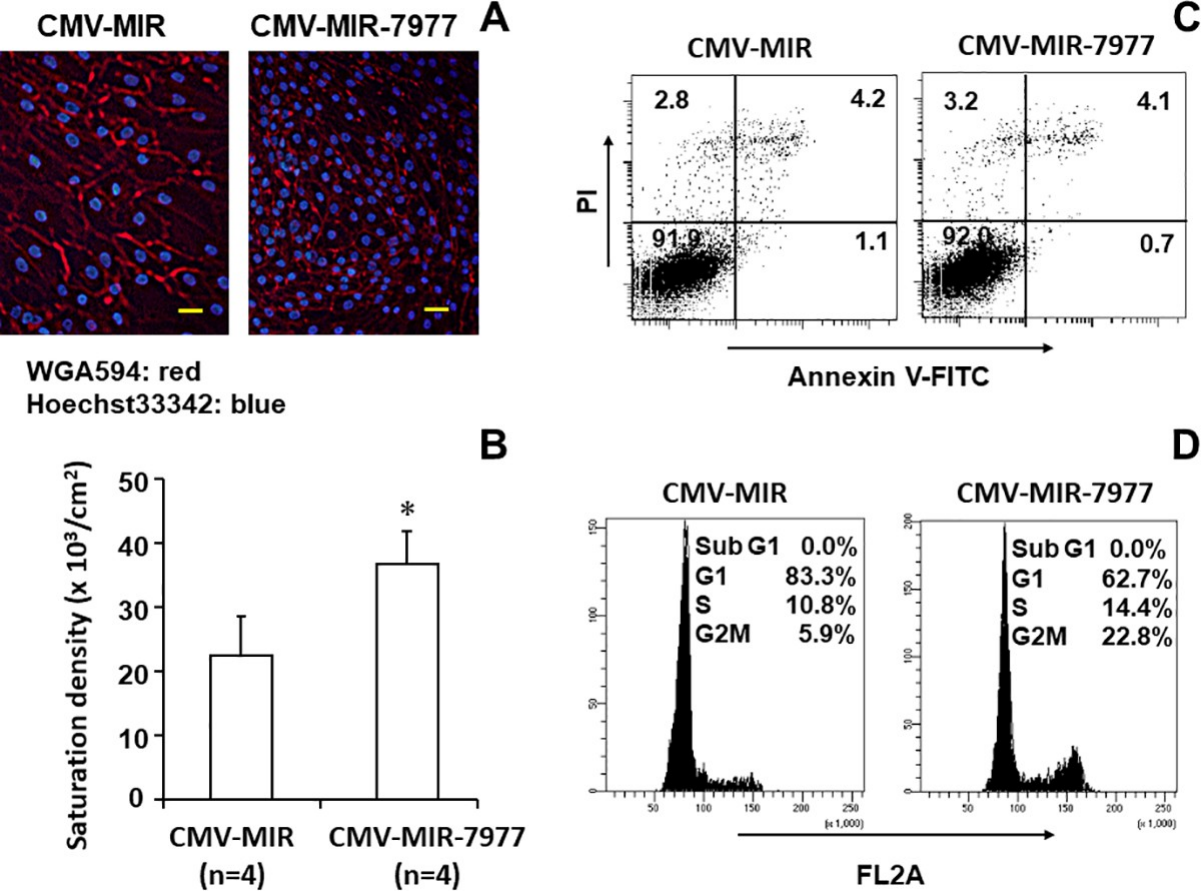
Alteration of cell function after miR-7977 transfer. (A) pCMV-MIR- or pCMV-7977-transduced HTS-5 cells were visualized under a fluorescence microscope. Cell membrane was stained with WGA594 and nucleus was stained with Hoechst33342. Scale bar = 50 μm. (B) Saturation density was evaluated when HTS-5 reached confluence. pCMV-MIR-transduced HTS-5 vs. pCMV-7977-transduced HTS-5, *p = 0.00265. Transduced cells were selected with 175 μm Geneticin (G418) before analysis. (C) Annexin V/PI assay. The X-axis indicates Annexin V-FITC and Y-axis indicates PI. (D) Cell cycle analysis. G1 84.2±2.46 vs. 61.4±1.10, p<0.0018 (n = 3); S 10.0±2.07 vs. 14.0±0.94, p<0.0035; G2M 5.63±0.60 vs. 24.5±1.26, p = 0.00042.

We confirmed the function of miR-7977-transduced MSCs. It has been shown that Hippo signaling is involved in contact inhibition of cell growth [17]. Hence, we observed the density of cells when confluence was reached. As shown in Fig 5A and S6 Fig, the density of miR-7977-transduced HTS-5 appeared to be higher than that of control-transduced HTS-5. In fact, the number of miR-7977-transduced HTS-5 cells was significantly higher than that of control-transduced HTS-5 cells (Fig 5B). The annexin V/PI assay revealed that the numbers of apoptotic cells in the two groups were identical (Fig 5C). A greater percentage of cells were entering the cell cycle in miR-7977-transduced HTS-5 cells than in control-transduced HTS-5 cells (Fig 5D). These results strongly suggest that miR-7977 inactivates contact inhibition probably due to inhibition of the Hippo signaling pathway.

## Discussion

In the present study, we revealed that transfer of miR-7977 reduced the expression of STK4 and inhibited the Hippo-YAP1 signaling pathway in MSCs. Moreover, miR-7977 inactivated contact inhibition and subsequently promoted proliferation of the HTS-5 mesenchymal cell line.

Recent advance of high throughput sequencing technology revealed that multiple gene mutations in hematopoietic stem/progenitor cells could be involved in the development of MDS/AML [18]. However, little is known how genetic damage could be induced even in younger patients. Recently, it has been shown by our group and others that abnormal protein expression, such as down regulation of Dicer1 and Drosha expression (impaired miRNA biogenesis) [3], hedgehog-interacting protein [1] or aurora kinase A/B [19], occurs in MSCs derived from MDS and AML patients. Moreover, studies using mesenchymal cell-specific knockout mice such as Dicer1 [4] or β-catenin [20], demonstrated developed dysplastic changes in hematopoietic cells, accumulation of genetic mutation in hematopoietic cells and eventually development of AML. These findings suggest that dysfunction of MSCs could be associated with the development of AML. Based on these findings, we focused on the abnormality of BM MSCs derived from AML.

The significance of the Hippo-YAP1 signaling pathway has recently been identified in myeloma cells [21–23]. However, the contribution of Hippo signaling to the pathophysiology of AML remains unclear although a few reports mentioned Hippo signaling in AML cells [21, 24]. Regarding MSCs in patients with AML, it has been demonstrated that deregulation of proteoglycans, adhesion molecules, expression of cytokines, metabolic and endocytosis pathways could be detected by whole-exome sequencing [25]. Importantly, the BM MSCs derived from AML exhibit higher clonogenic potential than healthy donor-derived BM MSCs [26]. This finding was consistent with our present study showing MSCs readily entry into cell cycle (Fig 5). Additionally, some of studies indicated that genetic variations of BM MSCs derived from AML by targeted deep sequencing [27]. However, the mechanism that induced abnormality and higher proliferative potential of BM MSC in AML is not clearly understood.

Recently, interaction between tumor cells and stromal cells has been increasingly focused on [28–30]. In this regard, we have recently indicated that high concentration of extracellular vesicles (EVs) including exosome carrying miRNA existed in BM interstitial fluid [2]. However, it is unclear whether EVs effect on AML cells, normal hematopoietic cells or microenvironments. One possibility was that EVs derived from BM MSCs effect on AML cells. In this regard, Barrera-Ramirez J et al. demonstrated that the profile of EV miRNAs from MSCs derived from in patients with AML was analyzed by RNA sequencing and found that some of miRNAs such as miR-26a-5p and miR-101-3p were significantly increased concomitant with reduction of EZH2 and GSK3β mRNA. These results suggested that EVs from MSCs could be associated with leukemogenesis [31]. In contrast, it has been recently demonstrated that EV miR-34c-5p from MSCs promotes senescence of AML stem cells, resulted in inhibition of leukemogenesis [32]. Thus, depending upon the components of EV miRNAs from BM MSCs, their effects could be quite diverse. Another possibility was that EVs released from AML cells effect on normal hematopoietic cells, endothelial cells and MSCs. Recently, it was shown that EV miR-150 and miR-155 targeting c-MYB directly suppresses the clonogenicities of normal hematopoietic progenitor cells [33]. Moreover, it was reported that EV miRNA derived from AML effect on endothelial cells and promote chemoresistance via vascular remodeling [34]. Furthermore, it was revealed that EV miRNA released from AML cells remodels MSCs into a leukemia growth-permissive and normal hematopoiesis-suppressive microenvironment [35]. Collectively, EV miRNAs in BM interstitial fluid have diverse function and various targets of cells.

Regarding our present study, the expression level of miR-7977 in leukemic cells was around 1000-fold higher than that in MSCs (S7 Fig). Therefore, we focused on the function of EV miR-7977 against BM MSCs. It has been shown that nuclear localized YAP1 could induce both apoptosis and cell cycle entry [21]. The reduction of STK4 restored the level of nuclear YAP1 and triggered cell death via p73 in myeloid malignancy [21]. In the present study, miR-7977-transduced mesenchymal cell line, HTS-5 cells did not show increased apoptosis. Conversely, miR-7977-transduced cells showed increased entry into the cell cycle via transcription factor, TEAD (Fig 4 and Fig 5). Hippo-YAP1 signaling was identified to be involved in contact inhibition and controlling organ size [17]. Hippo-YAP1 signaling could mainly controls the cell cycle for genetically normal cells via TEAD (Fig 4).

Finally, we previously reported that miR-7977-transduced MSCs showed reduction of normal hematopoiesis via reductions of SCF, ANGPT1 and JAG1 [2]. These factors may be essential for self-renewal of HSCs. Collectively, miR-7977 altered the function of hematopoietic niche so that the niche is unfavorable for normal HSCs. Further biological studies should be required to elucidate the precise miR-7977 function in BM microenvironment in vivo.

## Conclusions

The miR-7977 is an important factor that inhibits normal hematopoiesis via reduction of several factors in BM MSC and could contribute to propagate functionally-disturbed MSCs via inactivation of Hippo-YAP signaling pathway in AML.

## Supporting information

S1 Table Real-time SYBR Green PCR and miScript Primer assay, primer set IDs. (PDF)

S2 Table miRNA mimics and IDs. (PDF)

S3 Table Differentially expressed genes after miR-7977 transfer into BM MSCs. (PDF)

S4 Table Summary of results of gene set enrichment analysis (GSEA) showing NOM p-val <0.00000001. (PDF)

S1 Fig KEGG pathway map04130 regarding SNARE interactions in vesicular transport. The fold changes of expression of genes after control or miR-7977 transfer were superimposed on this pathway.
(TIF)

S2 Fig KEGG pathway map04962 regarding vasopressin-regulated water reabsorption. The fold changes of expression of genes after control or miR-7977 transfer were superimposed on this pathway. This pathway mainly worked in the kidney. The role of this pathway in BM is now unclear.
(TIF)

S3 Fig Immunoblot analysis of Hippo signaling pathway after transfer of control siRNA or miR-7977 into HTS-5. (A) Anti-STK4 Ab, (B) anti-YAP1 Ab, and (C) anti-phospho-YAP1 Ser127 Ab were used. As internal standard, (D) anti-β-actin Ab was used. Molecular size was indicated at the left side of images.
(TIF)

S4 Fig The reduction of NF2 mRNA after miR-7977 transfer. (A) The expression level of NF2 obtained from the microarray is shown. Control-transduced MSCs vs. miR-7977-transduced MSCs, *p<0.05. (B) qRT-PCR for NF2 was conducted after transduction of control or 5 nM miR-7977. Each bar represents the mean and standard deviation (n = 5). (C) Immunoblot analysis of NF2 and internal standard (β-Actin) in control-transduced MSCs and miR-7977-transduced MSCs.
(TIF)

S5 Fig The original picture of Fig 4 indicating the effect of miR-7977 transfer on nuclear localization of YAP1. Left panel: control siRNA transfer. Right panel: miR-7977 transfer. Scale = 20 μm as indicated left panel.
(TIF)

S6 Fig The original picture of Fig 5. pCMV-MIR- or pCMV-7977-transduced HTS-5 cells were visualized under a fluorescence microscope. Cell membrane was stained with WGA594 and nucleus was stained with Hoechst33342. Scale bar (50 μm) was indicated on the lower panel.
(TIF)

S7 Fig The expression level of miR-7977 in leukemic cell lines, MSCs and HTS-5. Y-axis indicates that miR-7977 levels in several types of cells. As internal standard, 18S was used in this analysis because some of internal standard of small RNA levels were quite differ in hematopoietic cells and MSCs.
(TIF)
