# Supplementary material for: miR-7977 inhibits the Hippo-YAP signaling pathway in bone marrow mesenchymal stromal cells
Source: PLoS One. 2019 Mar 5;14(3):e0213220. doi: 10.1371/journal.pone.0213220 (PMC6400381; doi:10.1371/journal.pone.0213220)
Supplement: S1 Table — (PDF) [file pone.0213220.s001.pdf]

S1 Table. Real-time SYBR Green PCR and miScript Primer assay, primer set IDs

**Gene symbol**	**Primer set ID**
STK4	PPH05582A
NF2	PPH00203A
18S rRNA	PPH05666E
GAPDH	PPH00150F
human miR-7977	MS00048930
