# Supplementary material for: miR-7977 inhibits the Hippo-YAP signaling pathway in bone marrow mesenchymal stromal cells
Source: PLoS One. 2019 Mar 5;14(3):e0213220. doi: 10.1371/journal.pone.0213220 (PMC6400381; doi:10.1371/journal.pone.0213220)
Supplement: S2 Table — (PDF) [file pone.0213220.s002.pdf]

S2 Table. miRNA mimics and IDs

**miRNA mimic**	**ID**
Syn-hsa-miR-7977 miScript miRNA Mimic	MSY0031180
AllStars Negative Control siRNA	1027280
