# Supplementary material for: miR-7977 inhibits the Hippo-YAP signaling pathway in bone marrow mesenchymal stromal cells
Source: PLoS One. 2019 Mar 5;14(3):e0213220. doi: 10.1371/journal.pone.0213220 (PMC6400381; doi:10.1371/journal.pone.0213220)
Supplement: S3 Table — (PDF) [file pone.0213220.s003.pdf]

S3Table. Differentially expressed genes after miR-7977 transfer into BM MSCs

	logFC	AveExpr	P-value	Q-value
SDF2	-1.659481072	7.142454246	9.55E-07	0.025482874
VAMP3	-1.447390282	8.745490871	3.79E-06	0.03803031
MT1F	-1.137358443	4.331079081	7.14E-06	0.03803031
CXCL16	-0.972693062	7.381105007	8.04E-06	0.03803031
TAS2R50	-0.924934641	3.746830481	5.58E-05	0.065984866
LANCL1	-0.920091798	6.249549954	4.59E-05	0.061183796
ECE1	-0.911876499	7.833259493	2.39E-05	0.059472048
NSUN2	-0.908902053	6.669035044	6.64E-05	0.068143277
PLXND1	-0.898737249	7.532818324	1.48E-05	0.049450064
FANCI	-0.876018515	4.445044695	1.19E-05	0.045313607
PCBP1	-0.840979349	8.996033933	3.23E-05	0.061183796
FRRS1	-0.83044258	5.700080917	6.56E-05	0.068143277
CLDND1	-0.816405302	7.040254786	8.46E-05	0.06963848
SDCBP	-0.809988744	5.028508616	8.22E-05	0.06963848
MT1A	-0.80524869	3.295400994	0.002244813	0.199591915
AAED1	-0.778115634	6.342777934	2.98E-05	0.061183796
ITGB3	-0.775634686	6.983356729	9.87E-05	0.06963848
YIPF4	-0.774605446	7.445172495	0.000242448	0.102694019
MEGF9	-0.769401783	7.120196686	9.60E-05	0.06963848
PABPC3	-0.751596826	1.564802041	0.00068019	0.161189374
CIART	-0.74310977	6.037710972	0.000950513	0.170510169
HMGCR	-0.7297727	6.629227593	4.15E-05	0.061183796
PNP	-0.716210566	7.341914894	4.57E-05	0.061183796
ZNF222	-0.704818116	3.276275634	0.001412912	0.184821329
FKSG29	-0.703276499	5.032871999	7.84E-05	0.06963848
HGF	-0.70121155	6.811147232	5.69E-05	0.065984866
ATP9A	-0.698670343	6.853716607	4.15E-05	0.061183796
NDST1	-0.68053777	8.739102447	3.63E-05	0.061183796
RP2	-0.672373875	7.168207849	0.000100158	0.06963848
PIK3IP1	-0.66792009	5.509818935	0.000210829	0.090741526
OR10J5	-0.66166734	3.372834525	0.000139988	0.079480375
SERPINB3	-0.656134784	1.993465202	0.000119016	0.072544169
DNAJC12	-0.654620178	5.040384169	9.16E-05	0.06963848
NF2	-0.6531436	7.52374098	0.000106996	0.06963848
ZNF383	-0.653039018	5.041864221	0.000119616	0.072544169
GPSM2	-0.652142871	5.403529902	0.000247407	0.103157185
CLINT1	-0.651000678	8.800244612	0.000596018	0.160653892
APOL6	-0.648927797	6.974665843	0.001983355	0.193867488
HIST1H2AB	-0.642602304	3.359560151	0.002422078	0.201810458
METTL7A	-0.640434571	6.413171987	0.00147008	0.185276887
OR1F1	-0.640329261	3.183574574	0.000203383	0.09072911
DENND6A	-0.637636729	6.854801919	0.000452772	0.138439839
RPIA	-0.633275252	4.665016091	0.001394033	0.184157322
AP1M2	-0.623118049	4.552672804	0.002266643	0.199591915
ASB6	-0.612251299	7.268200051	0.000185155	0.08983845
SLC16A4	-0.610890491	7.663385289	7.87E-05	0.06963848
S1PR2	-0.609849813	6.57430781	0.000837658	0.170510169
STEAP4	-0.60379663	6.73797931	0.000336205	0.118194845
CNEP1R1	-0.603168295	7.528286526	0.001242588	0.180436022
YY1AP1	-0.600376236	5.31740838	0.000414113	0.134010698
STK4	-0.599839137	6.554299136	0.002490387	0.201810458
MT1X	-0.598745625	8.795565863	0.000166374	0.088793563
PNO1	-0.596594917	7.094158216	0.00020454	0.09072911
PPIP5K2	-0.594451173	7.268741732	0.000296967	0.116537755
