# Supplementary material for: miR-7977 inhibits the Hippo-YAP signaling pathway in bone marrow mesenchymal stromal cells
Source: PLoS One. 2019 Mar 5;14(3):e0213220. doi: 10.1371/journal.pone.0213220 (PMC6400381; doi:10.1371/journal.pone.0213220)
Supplement: S4 Table — (PDF) [file pone.0213220.s004.pdf]

**S4 Table. Summary of results of gene set enrichment analysis (GSEA) showing NOM p-val <0.00000001**

| GENE SET NAME | NES | NOM p-val | FDR q-val |
|---|---|---|---|
| YAP1_UP | 1.5068 | 0.0000 | 0.2027 |
| KEGG_SNARE_INTERACTIONS_IN_VESICULAR_TRANSPORT | -1.6243 | 0.0000 | 0.2333 |
| KEGG_VASOPRESSIN_REGULATED_WATER_REABSORPTION | -1.8425 | 0.0000 | 0.0520 |
| SRC_UP.V1_DN | 1.2965 | 0.0000 | 0.3038 |
| PRC2_EZH2_UP.V1_UP | 1.3032 | 0.0000 | 0.3095 |
| SIRNA_EIF4GI_UP | 1.3479 | 0.0000 | 0.3199 |
| PDGF_ERK_DN.V1_DN | 1.2752 | 0.0000 | 0.3282 |
| CAMP_UP.V1_DN | 1.3580 | 0.0000 | 0.3754 |
| VEGF_A_UP.V1_DN | 1.3679 | 0.0000 | 0.4173 |
| EGFR_UP.V1_DN | 1.3733 | 0.0000 | 0.4850 |
| RPS14_DN.V1_DN | 1.4045 | 0.0000 | 0.5241 |
| HALLMARK_ALLOGRAFT_REJECTION | 1.2234 | 0.0000 | 0.5729 |
| HALLMARK_KRAS_SIGNALING_DN | 1.1877 | 0.0000 | 0.5787 |
| HALLMARK_P53_PATHWAY | 1.2366 | 0.0000 | 0.6539 |
| KEGG_NITROGEN_METABOLISM | 1.3506 | 0.0000 | 0.6925 |
| KEGG_REGULATION_OF_ACTIN_CYTOSKELETON | 1.2411 | 0.0000 | 0.7718 |
| KEGG_MAPK_SIGNALING_PATHWAY | 1.1059 | 0.0000 | 0.8035 |
| REACTOME_CELL_CELL_COMMUNICATION | 1.1958 | 0.0000 | 0.8686 |
| REACTOME_G_ALPHA_S_SIGNALLING_EVENTS | 1.2579 | 0.0000 | 0.8739 |
| REACTOME_GPCR_LIGAND_BINDING | 1.2632 | 0.0000 | 0.8776 |
| REACTOME_POTASSIUM_CHANNELS | 1.2369 | 0.0000 | 0.9003 |
| HALLMARK_APICAL_SURFACE | 1.4093 | 0.0000 | 0.9271 |
| REACTOME_LIPID_DIGESTION_MOBILIZATION_AND_TRANSPORT | 1.2775 | 0.0000 | 0.9490 |
| REACTOME_AMINO_ACID_TRANSPORT_ACROSS_THE_PLASMA_ MEMBRANE | 1.2905 | 0.0000 | 0.9924 |
| KEGG_HEDGEHOG_SIGNALING_PATHWAY | 1.4858 | 0.0000 | 1.0000 |
| KEGG_TIGHT_JUNCTION | 1.4713 | 0.0000 | 1.0000 |
| REACTOME_REGULATION_OF_KIT_SIGNALING | 1.5374 | 0.0000 | 1.0000 |
| REACTOME_METAL_ION_SLC_TRANSPORTERS | 1.4304 | 0.0000 | 1.0000 |
| REACTOME_AMINE_LIGAND_BINDING_RECEPTORS | 1.4156 | 0.0000 | 1.0000 |
| REACTOME_GLUCAGON_TYPE_LIGAND_RECEPTORS | 1.3641 | 0.0000 | 1.0000 |
| REACTOME_NCAM1_INTERACTIONS | 1.3211 | 0.0000 | 1.0000 |
| BIOCARTA_CYTOKINE_PATHWAY | 1.3085 | 0.0000 | 1.0000 |
| KEGG_FC_GAMMA_R_MEDIATED_PHAGOCYTOSIS | 1.3963 | 0.0000 | 1.0000 |
