# Supplementary figures and images for: miR-7977 inhibits the Hippo-YAP signaling pathway in bone marrow mesenchymal stromal cells
Source: PLoS One. 2019 Mar 5;14(3):e0213220. doi: 10.1371/journal.pone.0213220 (PMC6400381; doi:10.1371/journal.pone.0213220)

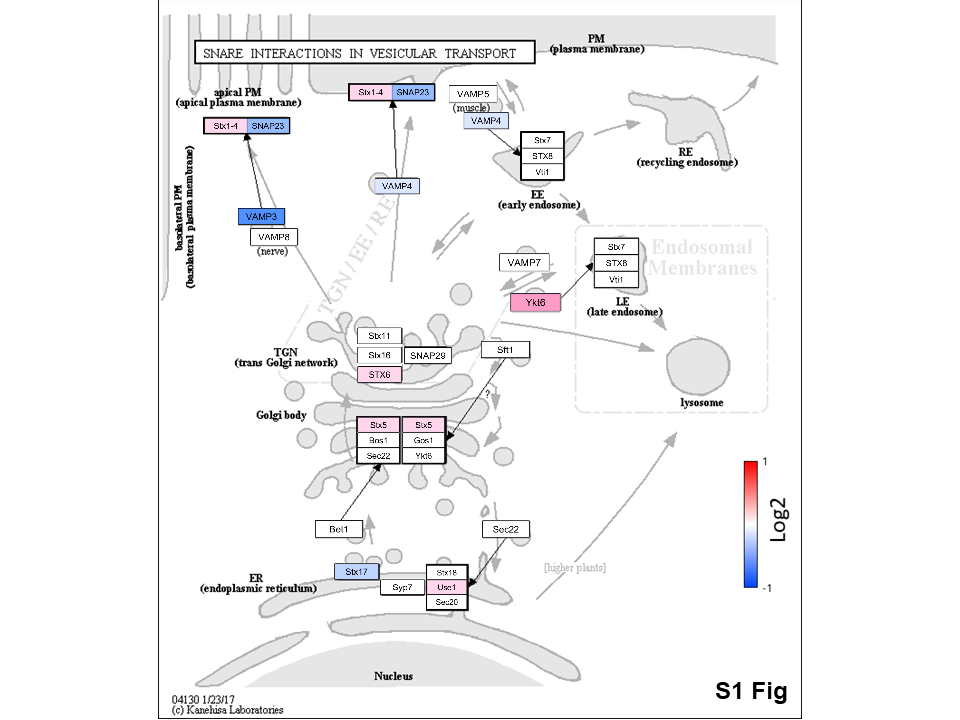

Supplement: S1 Fig — The fold changes of expression of genes after control or miR-7977 transfer were superimposed on this pathway. (TIF) [file pone.0213220.s005.TIF]

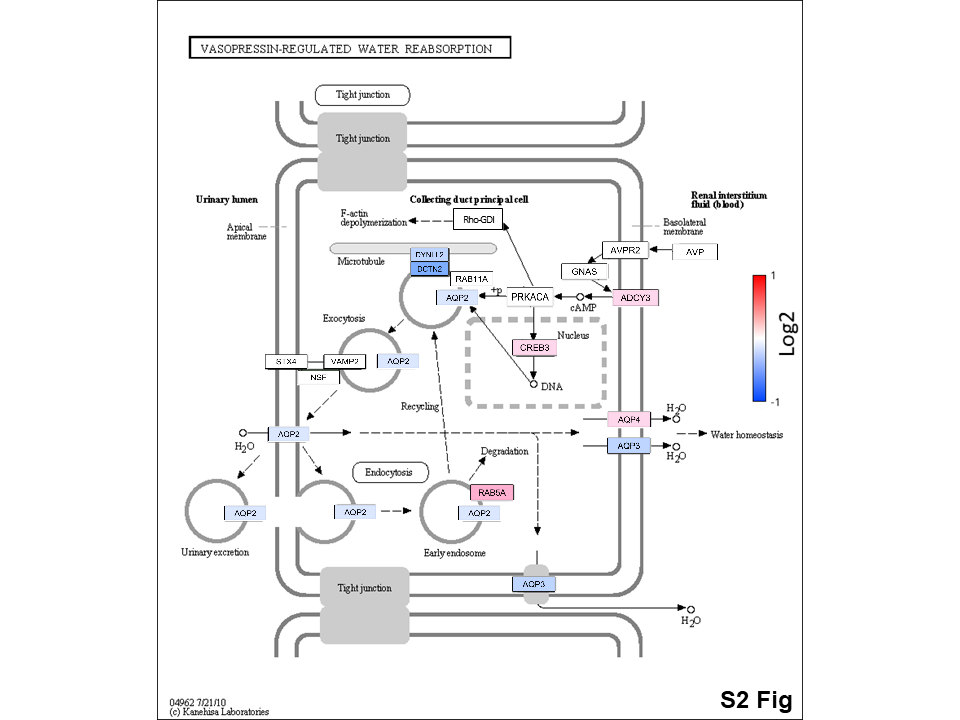

Supplement: S2 Fig — The fold changes of expression of genes after control or miR-7977 transfer were superimposed on this pathway. This pathway mainly worked in the kidney. The role of this pathway in BM is now unclear. (TIF) [file pone.0213220.s006.tif]

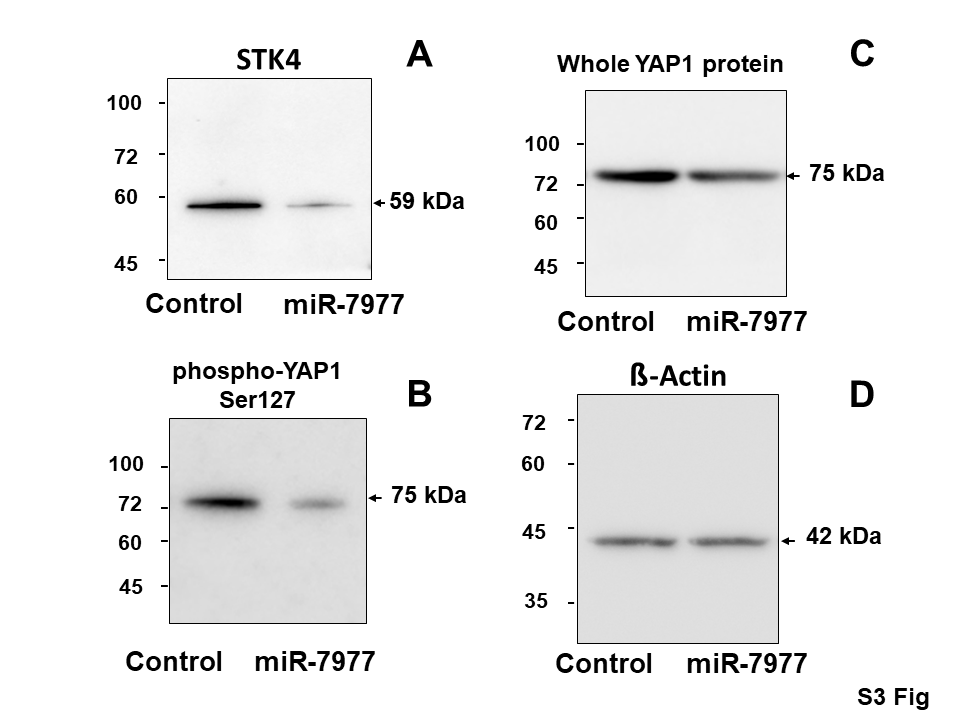

Supplement: S3 Fig — (A) Anti-STK4 Ab, (B) anti-YAP1 Ab, and (C) anti-phospho-YAP1 Ser127 Ab were used. As internal standard, (D) anti-β-actin Ab was used. Molecular size was indicated at the left side of images. (TIF) [file pone.0213220.s007.tif]

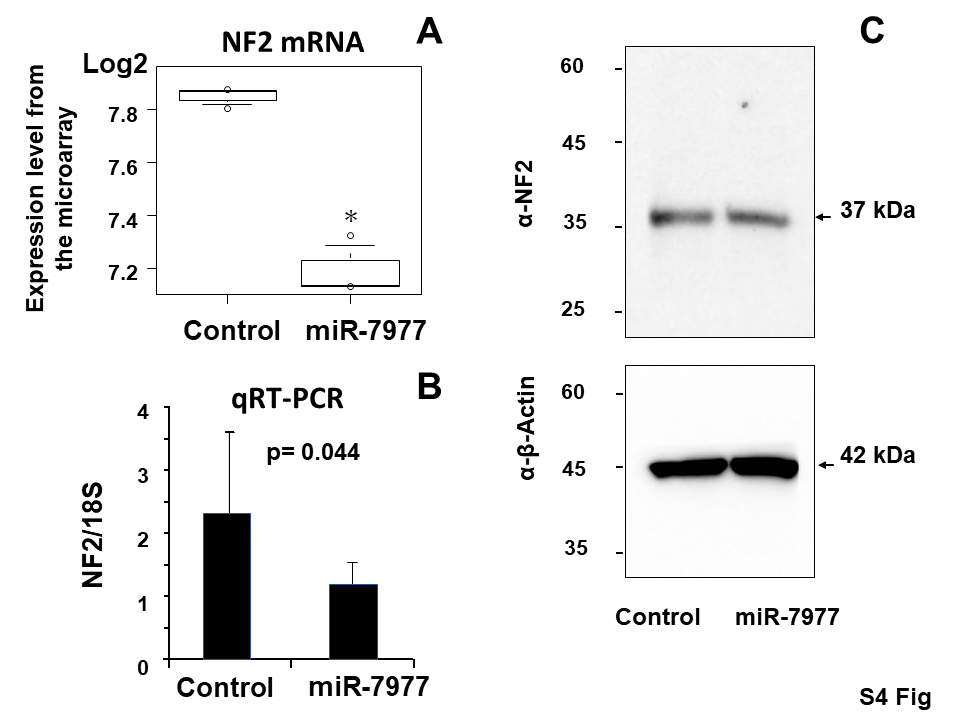

Supplement: S4 Fig — (A) The expression level of NF2 obtained from the microarray is shown. Control-transduced MSCs vs. miR-7977-transduced MSCs, *p<0.05. (B) qRT-PCR for NF2 was conducted after transduction of control or 5 nM miR-7977. Each bar represents the mean and standard deviation (n = 5). (C) Immunoblot analysis of NF2 and internal standard (β-Actin) in control-transduced MSCs and miR-7977-transduced MSCs. (TIF) [file pone.0213220.s008.tif]

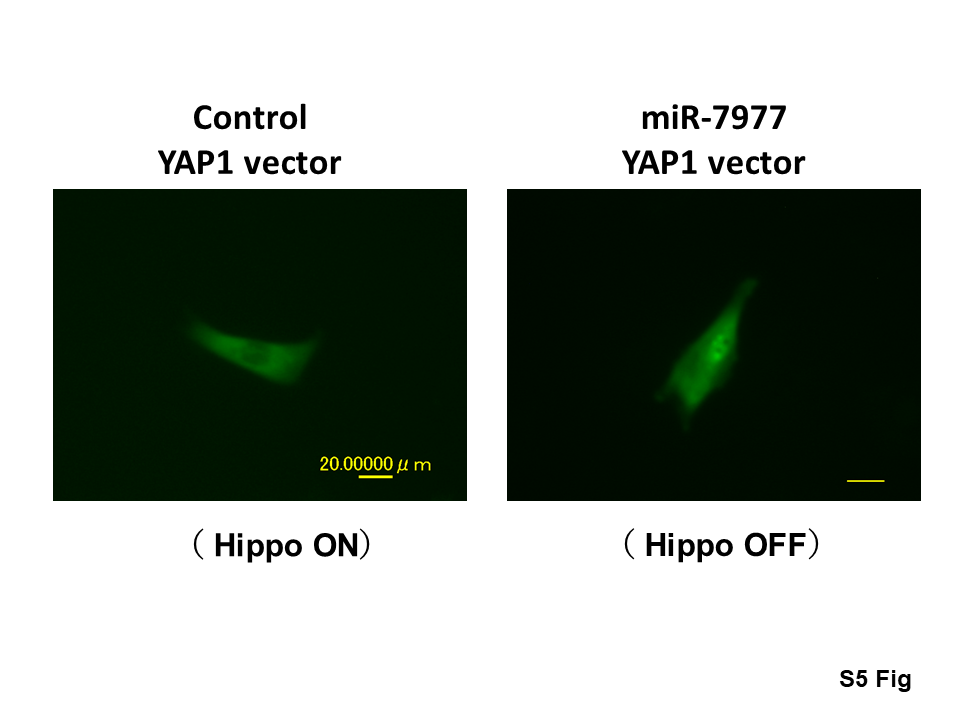

Supplement: S5 Fig — Left panel: control siRNA transfer. Right panel: miR-7977 transfer. Scale = 20 μm as indicated left panel. (TIF) [file pone.0213220.s009.tif]

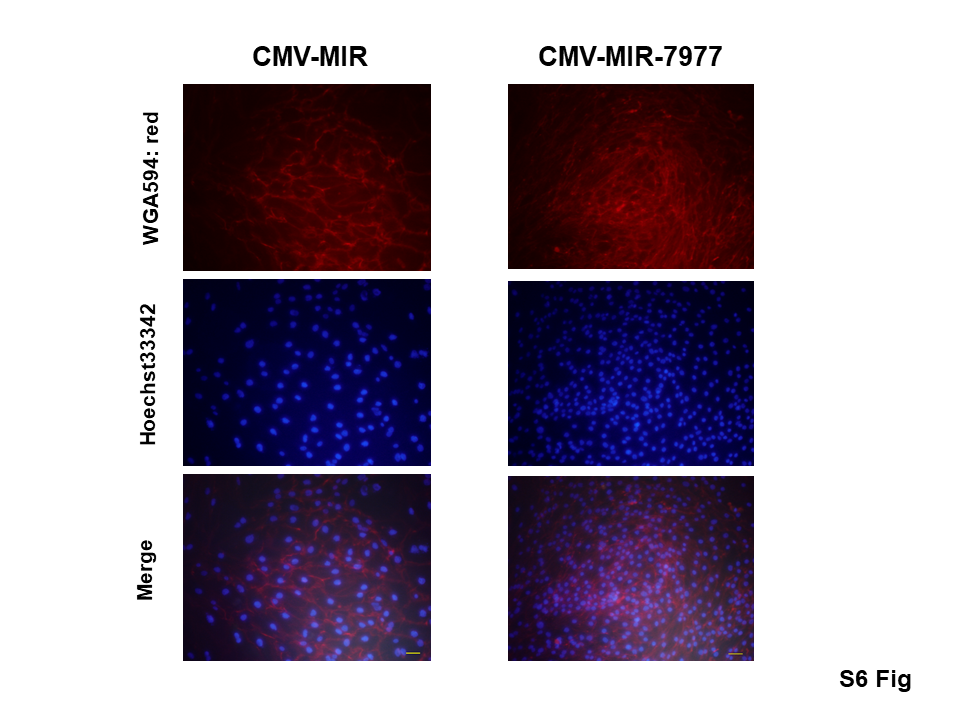

Supplement: S6 Fig — pCMV-MIR- or pCMV-7977-transduced HTS-5 cells were visualized under a fluorescence microscope. Cell membrane was stained with WGA594 and nucleus was stained with Hoechst33342. Scale bar (50 μm) was indicated on the lower panel. (TIF) [file pone.0213220.s010.tif]

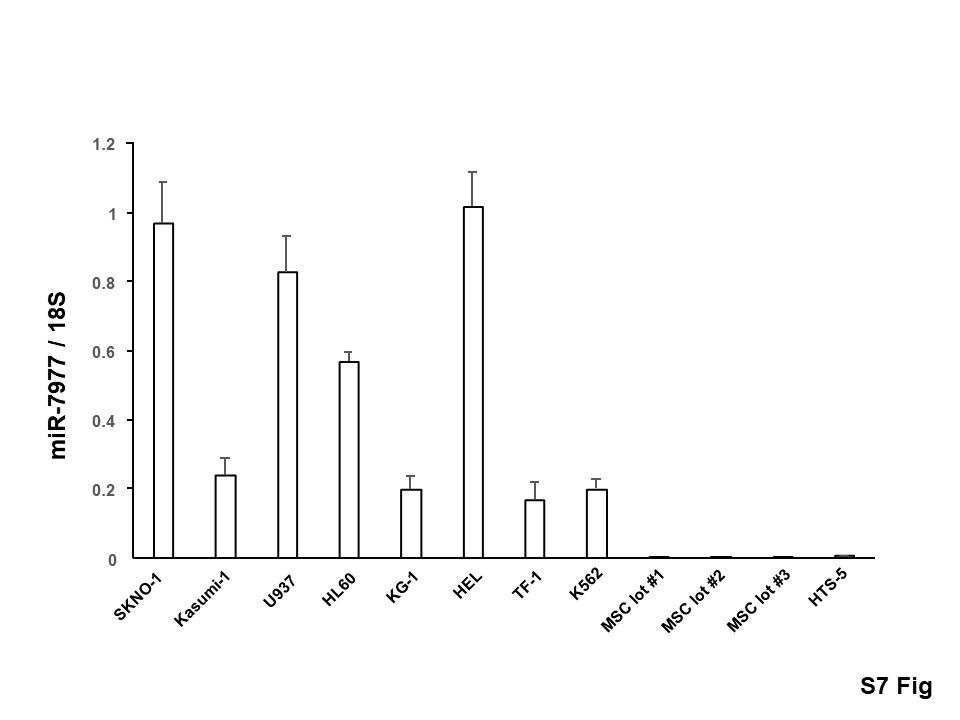

Supplement: S7 Fig — Y-axis indicates that miR-7977 levels in several types of cells. As internal standard, 18S was used in this analysis because some of internal standard of small RNA levels were quite differ in hematopoietic cells and MSCs. (TIF) [file pone.0213220.s011.tif]
